# Intravoxel Incoherent Motion MR Imaging: Comparison of Diffusion and Perfusion Characteristics for Differential Diagnosis of Soft Tissue Tumors

**DOI:** 10.1097/MD.0000000000001028

**Published:** 2015-06-26

**Authors:** Jun Du, Kun Li, Weisheng Zhang, Shaowu Wang, Qingwei Song, Ailian Liu, Yanwei Miao, Zhijin Lang, Lina Zhang, Minting Zheng

**Affiliations:** From the Department of Radiology, First Affiliated Hospital of Dalian Medical University (JD, WZ, QS, AL, YM, ZL, LZ, MZ); Department of Radiology, Second Affiliated Hospital of Dalian Medical University (SW); and Department of orthopedics, Second Affiliated Hospital of Dalian Medical University, Dalian, China (KL).

## Abstract

We used intravoxel incoherent motion (IVIM) magnetic resonance imaging (MRI) to explore the possibility of preoperative diagnosis of soft tissue tumors (STTs). This prospective study enrolled 23 patients. Conventional MRI and IVIM examinations were performed on a 3.0T MR imager. Eight (35%) hemangiomas, 11 (47%) benign soft tissue tumors excluding hemangiomas (BSTTEHs) and 4 soft tissue sarcomas (STSs) were assessed. The mean tumor size was about 1652.36 ± 233.66 mm^2^. Ten b values (0–800 s/mm^2^) were used to evaluate diffusion and perfusion characteristics of IVIM. IVIM parameters (ADC_standard_, ADC_slow_, ADC_fast_, and f) of STTs were measured and evaluated for differentiating hemangiomas, BSTTEHs, and STSs. ADC_slow_ and ADC_fast_ value were different for hemangiomas, BSTTEHs, and STSs separately (*P* < 0.001, *P* < 0.001, and *P* = 0.001). ADC_slow_, cut-off value smaller than 0.93 × 10^–3^ mm^2^/s, was the best parameter to differ STSs (0.689 ± 0.173 × 10^−3^ mm^2^/s) from hemangiomas (0.933 ± 0.237 × 10^−3^ mm^2^/s) and BSTTEHs (1.156 ± 0.120 × 10^−3^ mm^2^/s) (*P* = 0.001). ADC_slow_ (0.93 × 10^−3^ mm^2^/s <cut-off value <0.96 × 10^−3^ mm^2^/s) was used to distinguish hemangiomas from BSTTs. There were significant difference among hemangiomas, BSTTEHs, and STSs (*P* *=* 0.014, *P* *=* 0.036, *P* < 0.001). The ADC_standard_, ADC_fast_, and f value were different (*P* *<* 0.05) for STSs (1.009 ± 0.177 × 10^−3^ mm^2^/s, 15.700 ± 1.992 × 10^−3^ mm^2^/s, 0.503 ± 0.068), hemangiomas (1.505 ± 0.226 × 10^−3^ mm^2^/s, 11.675 ± 0.456 × 10^−3^ mm^2^/s, 0.682 ± 0.060), and BSTTEHs (1.555 ± 0.176 × 10^−3^ mm^2^/s, 11.727 ± 0.686 × 10^−3^ mm^2^/s, 0.675 ± 0.054). And there was no significant difference for these 3 parameters between hemangiomas and BSTTEHs (*P* *=* 0.584, 0.907, and 0.798). IVIM may be of significant value for differential diagnosing hemangiomas, BSTTEHs, and STSs.

## INTRODUCTION

Soft tissue tumors (STTs), putatively mesenchymal origin, are sporadic without a known pathogenesis or established risk factors. There is a rising incidence in recent years for benign soft tissue tumors (BSTTs) more than 300/100,000 and malignant ones around 5/100,000 per year.^1^ In addition, STTs represent a striking range of morphologic diversity and multiple individual clinical symptoms among different types.^2^ Differential diagnosis of benign tumors and malignant ones should be made for further treatment in clinic. Soft tissue sarcomas (STSs), as the main malignant tumors, occur at any age and account for only 21% of all pediatric solid malignant cancers and less than 1% of all adult solid malignant cancers.^3^ It is difficult to make a prospectively preoperative diagnosis of different subtypes of STTs due to their complexity and rarity.

Magnetic resonance imaging (MRI) is considered the first choice of imaging modality to evaluate STTs.^4^ However, there still are lots of problems need to be dealt with. For example, BSTTs may achieve dedifferentiation and exhibit hybrid histologic characteristics of malignant propensity. In addition, hemangiomas, accounting for 7% of BSTTs,^5^ show diffuse growth and easy to relapse, which is the main cause of clinical misdiagnosis as malignant tumor. It is reported that MR imaging cannot reliably distinguish benign and malignant lesions when radiologic evaluation is nonspecific.^6^ The inhomogeneity of lesions on T_2_, the change from homogeneity on T_1_ to inhomogeneity on T_2_ sequence, and the involvement of bone or neurovascular structures are features that may be helpful in differential diagnosing benign from malignant soft tissue masses, while whether MRI can be used to differentiate benign from malignant ones is still controversial.^7^

Intravoxel incoherent motion (IVIM) imaging is an extension of diffusion weighted imaging (DWI) that integrates the apparent diffusion coefficient (ADC) contains both perfusion and diffusion terms.^8^ Diffusion and perfusion are physically and biologically different phenomena.^9^ The diffusion property of tumor tissues largely depends on cell density, which may also be predictive features of malignancy in some types of tumors. Perfusion is an important phenomenon of many physiological or pathological processes.^8^ It is reported that IVIM imaging may be helpful for differentiating benign and malignant salivary gland tumors.^10^ Therefore, to estimate these 2 distinctive phenomena in tumor tissues may be helpful in the diagnosis of STTs before surgery.

The purpose of the present study was to assess the IVIM parameters for differential diagnosing hemangiomas, benign soft tissue tumors excluding hemangiomas (BSTTEHs), and STSs.

## PATIENTS AND METHODS

### Patients

The study was approved by the Institutional Ethics Committee of First Affiliated Hospital of Dalian Medical University (Dalian, China). Thirty-one consecutive patients were enrolled in this study and inspected conventional MR and IVIM-MR examination with 3.0-T MR imager from October 2013 to October 2014. Eight of these patients were excluded for further analysis: 4 patients did not receive surgery and there were no pathological results available; 1 tumor in the upper limb was close to artery and the quality of image was very poor; and for other 3 patients, there were no sufficient regions of interest (ROI) areas for IVIM analysis. As a result, only 23 STTs were further analyzed. Primary sites and clinical data of these tumors (11 female, 12 male; average age, 44 years; age range 16–85 years) were described in Table 1.

**TABLE 1:**
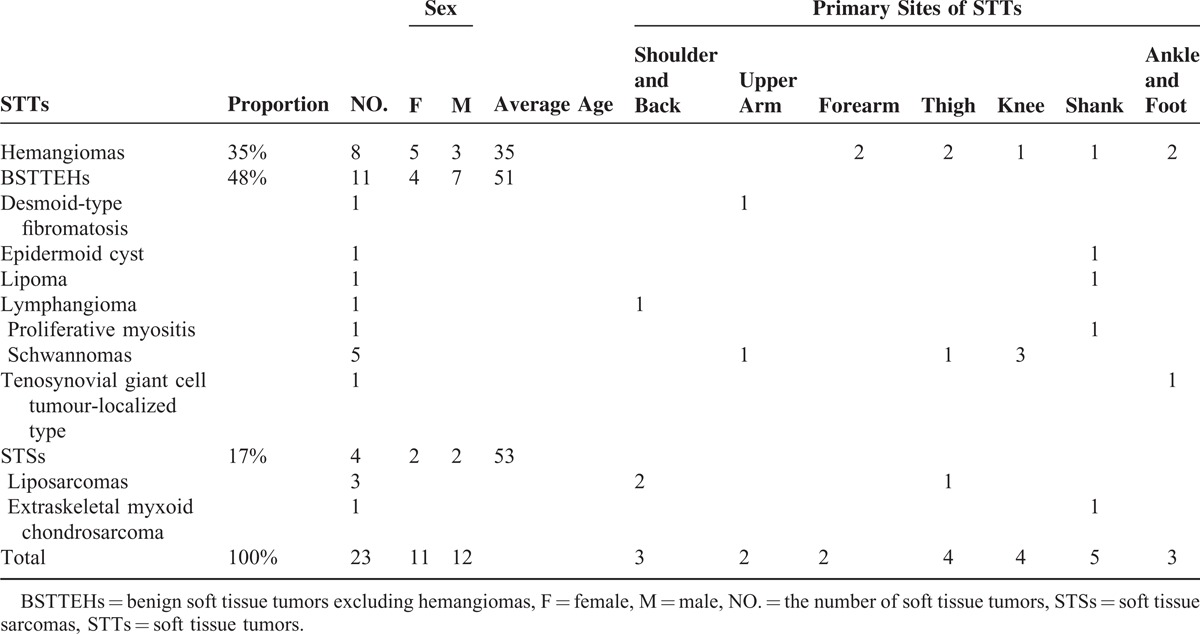
Primary Sites and Clinical Data of 23 Soft Tissue Tumors

### Conventional MR Imaging

MRI was performed using a 3.0-T MR imager (Signa, HDxt, 3.0 T; General Electric Healthcare, Milwaukee, WI) with surface coil (limbs with 3 inch coil, shoulder with shoulder coil or soft coil, and torso and hips with body coil). The conventional MR scanning sequences included spin echo (SE) T1WI (repetition time/echo time [TR/TE], 650 ms/min full; reconstruction matrix size, 288 × 224; slice thickness/slice spacing, 4–6 mm/0–1 mm), fast spin echo (FSE) T_2_WI (TR/TE, 3975 ms/68 ms; number of signals acquired, 4; reconstruction matrix size, 256 × 224; slice thickness/slice spacing, 4–6 mm/0–1 mm), fat suppression T_2_WI and PDWI (TR/TE, 2000 ms/42 ms; number of signals acquired, 3; reconstruction matrix size, 256 × 224; slice thickness/slice spacing, 4–6 mm/0–1 mm). If necessary, fat suppression T_1_WI was performed. Field of view (FOV), which could be changed according to the tumors’ size, was 15 to 40 cm with slice thickness 4 to 6 mm and space 0 to 1 mm.

### IVIM MR Imaging

IVIM scan was performed using spin-echo echo-planar imaging (SE-EPI) sequence (TR/TE, 8000 ms/min; NEX, 8; reconstruction matrix size, 128 × 128; slice thickness, slice spacing, and FOV similar to conventional scan; 10 b values [0, 10, 20, 30, 50, 100, 200, 300, 500, and 800 s/mm^2^]). In order to eliminate the influence of the anisotropy for the IVIM signal and the numerical measurement, 3 in 1 was applied. All patients were fixed on bed to prevent the influence of movement.

Based on IVIM theory, the bi-exponential model was expressed by the following equation:^11^ 
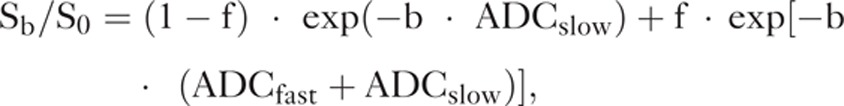


where S_b_ is the signal intensity in the pixel with diffusion gradient b, S_0_ the signal intensity in the pixel without diffusion gradient (b of 0 s/mm^2^), f the fractional perfusion related to the microcirculation, ADC_slow_ the true diffusion coefficient as reflected by pure molecular diffusion, and ADC_fast_ is the pseudo-diffusion coefficient representing perfusion-related diffusion or incoherent microcirculation.

### Dynamic Contrast-Enhanced MR Imaging

Dynamic contrast-enhanced MR imaging was performed using 2-dimensional fast spoiled gradient echo sequence (2D FSPGR). The contrast agent gadolinium-DTPA (Magnevist, Berlex, New Jersey) was injected as 0.l mmol/kg, and 2.5 mL/s. And 10 mL saline was additionally injected at the same velocity.

### Regions of Interest (ROI)

ROI was placed on the parenchyma of each tumor in DWI. Large cystic or necrotic areas and large vessels were not included. The mean ROI area was about 15.0 ± 1.21 mm^2^. Three ROIs in each image and totally 9 ROIs in 3 consecutive images of IVIM were calculated for each patient. The different values (ADC_standard_, ADC_slow_, ADC_fast_, and f) of STTs were measured in GE-ADW 4.4 workstation.

### Statistical Analysis

Two independent-sample *t*-test was used for assessing ADC_slow_ and ADC_fast_ of hemangiomas, BSTTEHs, and STSs separately. One-way ANOVA and least significant difference (LSD) *t*-test were used for comparing IVIM parameters among the 3 types of STTs. Receiver operating characteristic (ROC) curves were generated with respective cut-off values determined to accommodate best diagnostic accuracy based on the Youden index. SPSS (version19.0, Chicago, IL) was used for statistical analysis. *P* values < 0.05 were considered significant for 2-tailed probability.

## RESULTS

In total, ROIs from 8 hemangiomas, 11 BSTTEHs, and 4 STSs were evaluated. The mean tumor size (maximum tumor areas measured using axial contrast-enhanced MR images) was 1652.36 ± 233.66 mm^2^. ADC_slow_ and ADC_fast_ value were different among hemangiomas, BSTTEHs, and STSs separately (*P* *<* 0.001, *P* < 0.001, and *P* = 0.001).

Each IVIM parameter (ADC_standard_, ADC_slow_, ADC_fast_, and f) for STTs was significant difference (*P* < 0.001, *P* = 0.001, *P* < 0.001, and *P* < 0.001) (Table 2, Figures 3–5). Multiple comparisons of IVIM parameters between each index are summarized in Table 3. The ADC_standard_, ADC_fast_, and f values of hemangiomas and STSs or BSTTEHs and STSs were significant difference (*P* < 0.05), while there was no significant difference for these 3 parameters between hemangiomas and BSTTEHs (*P* = 0.584, *P* = 0.907, and *P* = 0.798). The ADC_slow_ values between hemangiomas and BSTTEHs, hemangiomas and STSs, and also BSTTEHs and STSs were significant difference (*P* = 0.014, *P* = 0.036, and *P* < 0.001).

**TABLE 2:**
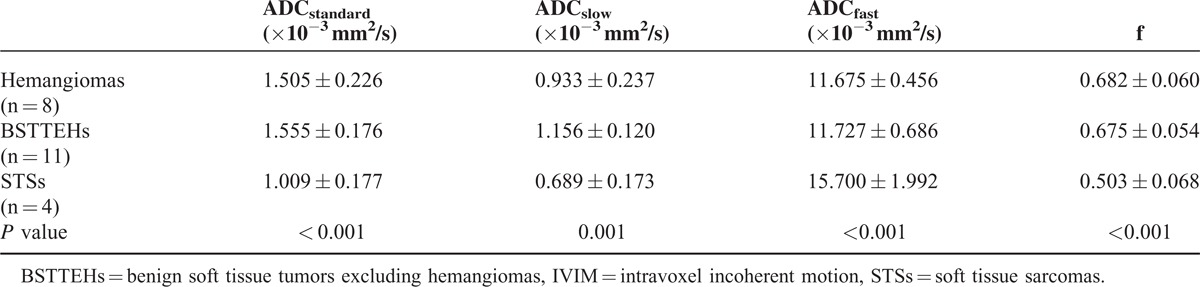
One-Way ANOVA Tests Differing IVIM Parameters Among Hemangiomas, BSTTEHs, and STSs

**TABLE 3:**
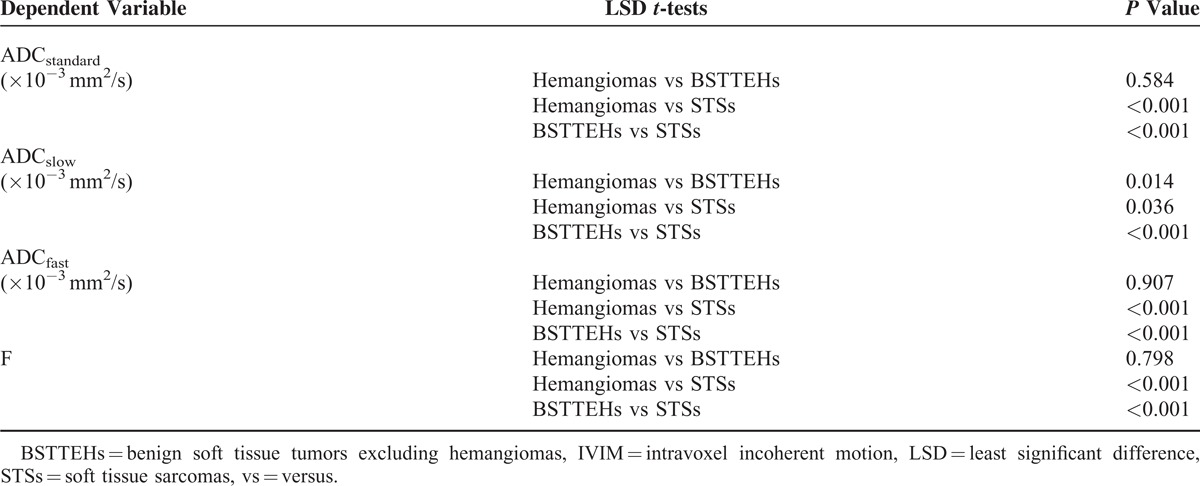
LSD *t*-tests of Multiple Comparison of IVIM Parameters Value Among Hemangiomas, BSTTEHs, and STSs

**FIGURE 1:**
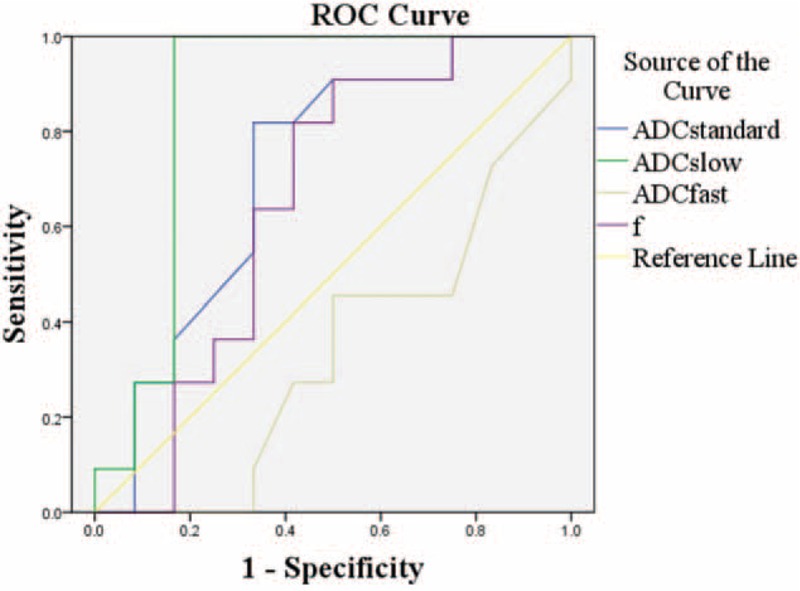
ROC curves of IVIM parameters for identifying soft tissue sarcoma from hemangioma and benign tumors.

**FIGURE 2:**
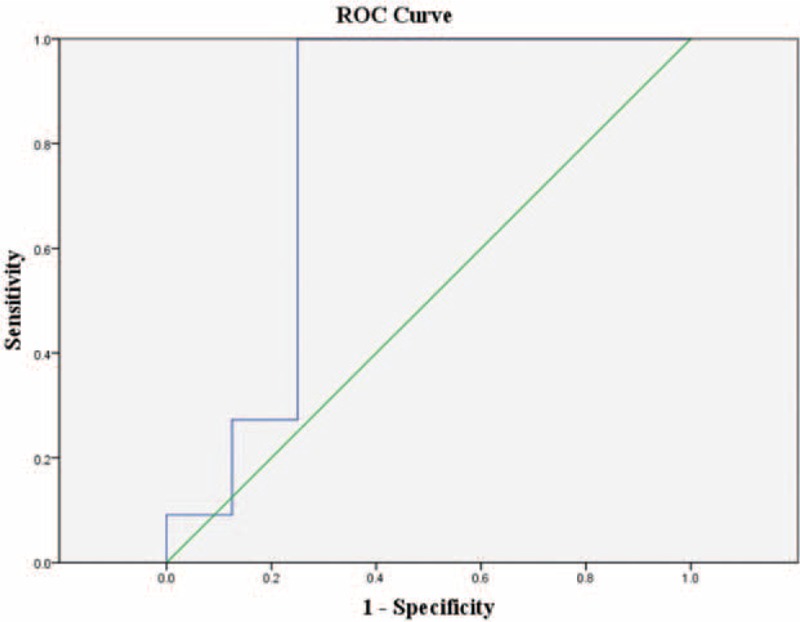
ROC curve of ADC_slow_ for discriminating hemangioma from benign tumors.

**FIGURE 3:**
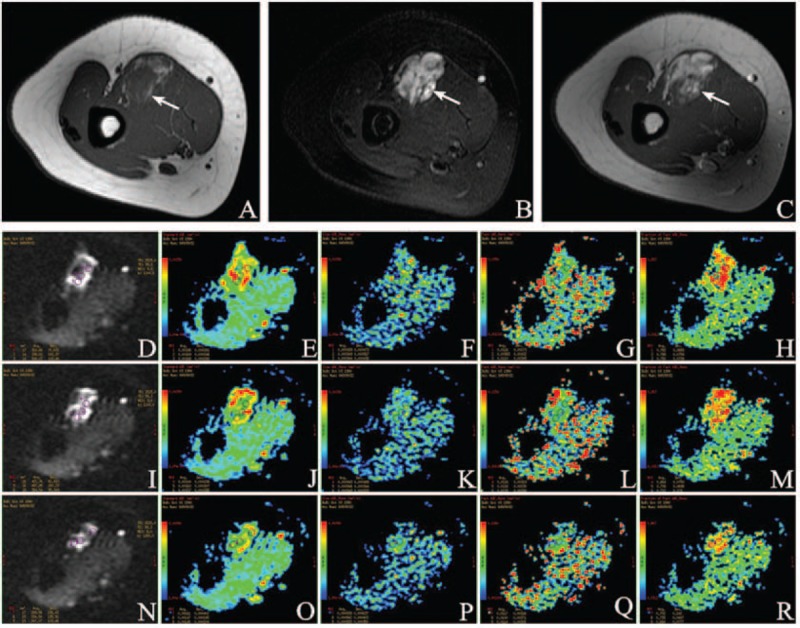
A 23-year-old female was diagnosed hemangioma (arrow) in the right brachialis. (A) Axial T_1_WI demonstrates a solid mass of intermediate signal intensity (SI) compared to adjacent muscles. (B) Axial T_2_WI shows a lobulated mass with high SI. (C) Contrast-enhanced T_1_WI image shows intense homogeneous enhancement with large internal vessels. Characteristic changes in intravoxel incoherent motion (IVIM) parameters: (D) Location maps of ROI_1_, ROI_2_, and ROI_3_ in the first sectional image. Images (E–H) show ADC_standard_, ADC_slow_, ADC_fast_, and f value separately. (I) Location maps of ROI_4_, ROI_5_, and ROI_6_ in the second sectional image. ADC_standard_, ADC_slow_, ADC_fast_, and f value were placed in (J–M) successively. (N) Location maps of ROI_7_, ROI_8_, and ROI_9_ in the third sectional image. ADC_standard_, ADC_slow_, ADC_fast_, and f value were put in (O–R) in proper order. Mean value of ADC_standard_ is 0.00160 ± 0.000288 mm^2^/s, ADC_slow_ 0.000943 ± 0.000448 mm^2^/s, ADC_fast_ 0.0111 ± 0.00319 mm^2^/s, and f 0.725 ± 0.0827 from ROI_1_ to ROI_9_, accordingly.

**FIGURE 4:**
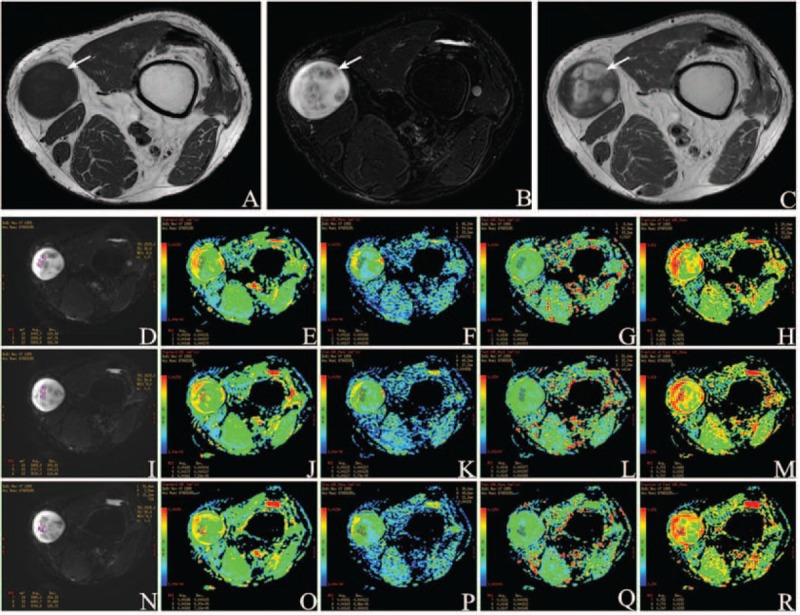
A 59-year-old male was diagnosed Schwannoma (arrow) in the subcutaneous fat of left knee between vastus medialis and sartorius. (A) Axial T_1_WI demonstrates a solid mass of intermediate signal intensity (SI). (B) Axial T_2_WI shows the ovoid mass with heterogeneously hyper SI. (C) Contrast-enhanced T_1_WI shows heterogeneously enhanced. Characteristic changes in IVIM parameters: (D) Location map of ROI_1_, ROI_2_, and ROI_3_ in the first sectional image. Images (E–H) separately show ADC_standard_, ADC_slow_, ADC_fast_, and f value. (I) Location map of ROI_4_, ROI_5_, and ROI_6_ in the second sectional image. ADC_standard_, ADC_slow_, ADC_fast_, and f value were placed in (J–M) successively. (N) Location maps of ROI_7_, ROI_8_, and ROI_9_ in the third sectional image. ADC_standard_, ADC_slow_, ADC_fast_, and f value were put in (O–R) in proper order. Mean value of ADC_standard_ is 0.00168 ± 0.000129 mm^2^/s, ADC_slow_ 0.00130 ± 0.000140 mm^2^/s, ADC_fast_ 0.0108 ± 0.000380 mm^2^/s, and f 0.714 ± 0.0307 from ROI_1_ to ROI_9_, accordingly.

**FIGURE 5:**
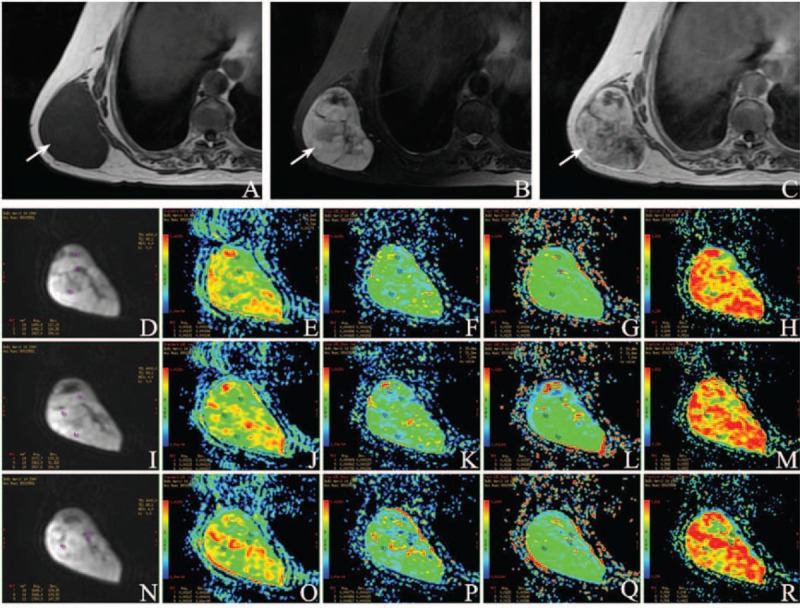
A 74-year-old female was diagnosed myxoid liposarcoma (arrow) in the subcutaneous fat of her right back. (A) Axial T_1_WI demonstrates a solid mass of low signal intensity (SI). (B) Axial T_2_WI shows a lobulated mass with high SI. (C) Contrast-enhanced T_1_WI shows heterogeneously enhanced solid tissues and thickened septa. Characteristic changes in IVIM parameters: (D) Location maps of ROI_1_, ROI_2_, and ROI_3_ in the first sectional image. Images (E–H) separately show ADC_standard_, ADC_slow_, ADC_fast_, and f value. (I) Location maps of ROI_4_, ROI_5_, and ROI_6_ in the second sectional image. ADC_standard_, ADC_slow_, ADC_fast_, and f value were placed in (J–M) successively. (N) Location maps of ROI_7_, ROI_8_, and ROI_9_ in the third sectional image. ADC_standard_, ADC_slow_, ADC_fast_, and f value were put in (O–R) in proper order. Mean value of ADC_standard_ is 0.00118 ± 0.000134 mm^2^/s, ADC_slow_ 0.000849 ± 0.000207 mm^2^/s, ADC_fast_ 0.0140 ± 0.00344 mm^2^/s, and f 0.562 ± 0.0631 from ROI_1_ to ROI_9_, accordingly.

The ROC curves and optimal cut-off values of IVIM parameters were used to identify STSs from STTs (Figure 1 and Table 4). The ADC_slow_ value was the most powerful parameter, with area under the curve of 0.86, followed by ADC_standard_ 0.72 and f 0.56. ADC_fast_ was the least meaningful one with area 0.34. The ROC curve and optimal cut-off value of ADC_slow_ were used to discriminate hemangiomas from BSTTs in Figure 2 and Table 5. The optimal cut-off value was between 0.93 × 10^−3^ and 0.96 × 10^−3^ mm^2^/s. Two steps to deal with STTs were indicated in Table 6. First step was to separate BSTTs (Figures 3 and 4) from STSs (Figure 5), and then to identify hemangiomas (Figure 3) from BSTTs.

**TABLE 4:**
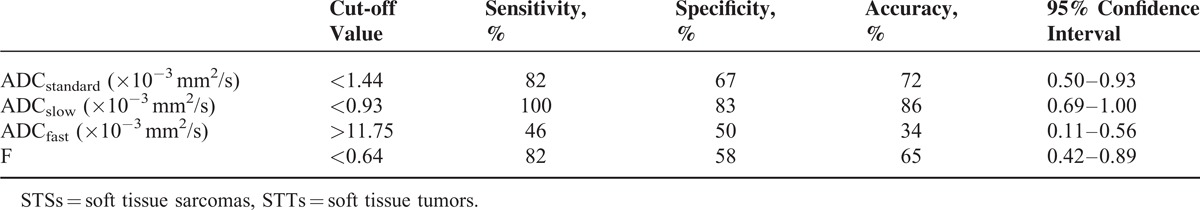
Diagnostic Characteristics of IVIM Parameters to Identify STSs From STTs Based on the Respective Cut-Off Values

**TABLE 5:**
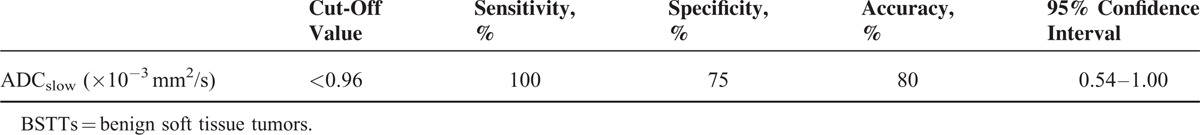
Diagnostic Characteristics of ADC_slow_ to Discriminate Hemangiomas from BSTTs Based on the Respective Cut-Off Values

**TABLE 6:**
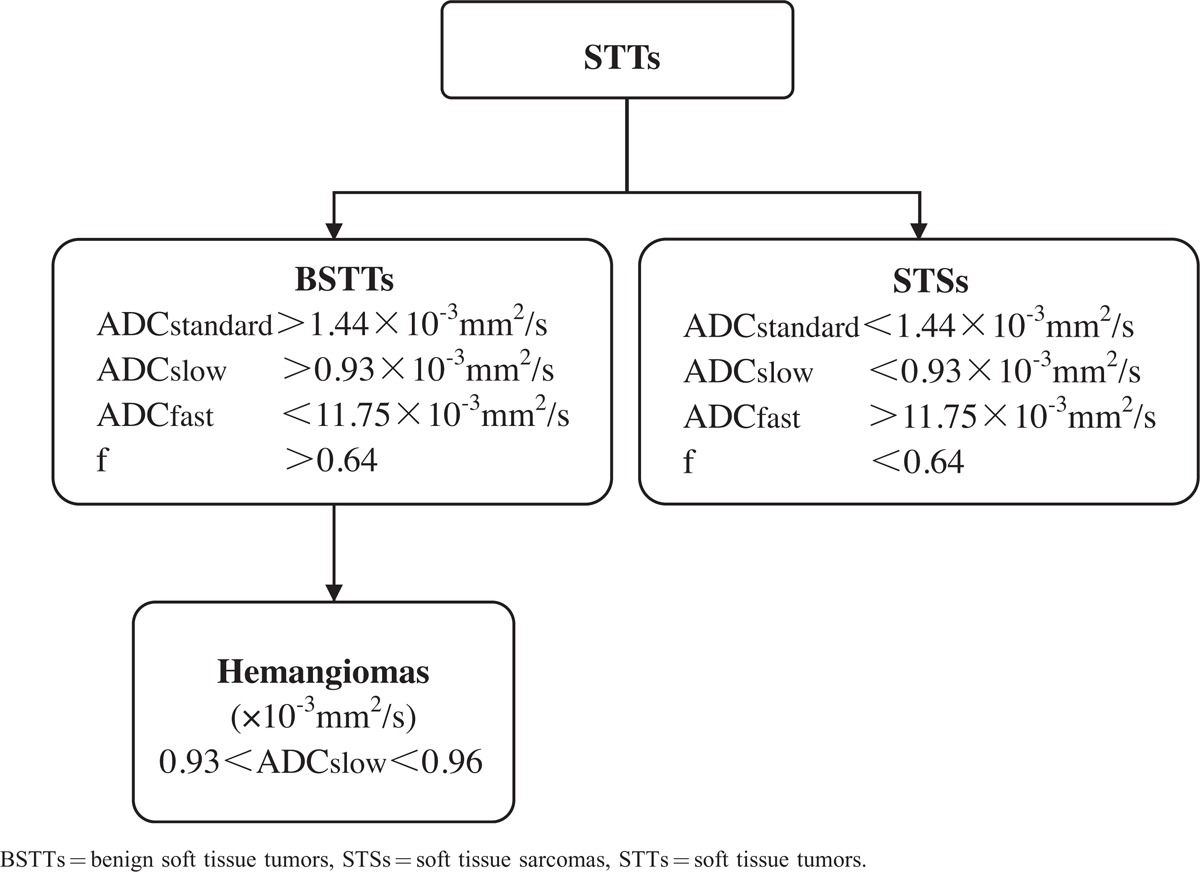
Two Steps to Deal with STTs: Firstly, Differentiating BSTTs and STSs, and Then Identifying Hemangiomas From BSTTs

## DISCUSSION

There are similar imaging characteristics (such as diffuse growth, easy to relapse, and high signal intensity on T_2_-weighted imaging/DW-MRI) for hemangiomas and malignant tumors. This is the main reason for radiological and clinical misdiagnosing hemangiomas as malignant tumors. So hemangiomas were classified out as a separate group in this study.

IVIM MR imaging has been utilized as an attractive noninvasive imaging technique with the potential for assessing both tissue perfusion and diffusion of STTs using a single DW imaging.^10,12^ And it shows a unique profile of microcirculation and pure molecular diffusion within tumors. Our study showed that the ADC_slow_ and ADC_fast_ values of hemangiomas, BSTTEHs, and STSs were significantly different, which suggested that IVIM imaging facilitates understanding of tumor tissue characteristics of perfusion and diffusion.

On the other hand, ADC_standard_ values of hemangiomas, BSTTEHs, and STSs were also significantly different. The ADC_standard_ of BSTTEHs was maximum (1.555 ± 0.176 × 10^−3^ mm^2^/s), while the ADC_standard_ of STSs was minimum (1.009 ± 0.177 × 10^−3^ mm^2^/s) due to active proliferative capacity of sarcomas. The limitation of water molecular diffusion of malignant tumors leads to the decrease of ADC value.^13^

In the bi-exponential model, ADC_slow_ (*P* = 0.014) was an effective parameter to distinguish hemangiomas from other benign tumors. ADC_slow_ with a high b value (>200 s/mm^2^) is the true diffusion coefficient of pure water in tumors with perfusion components removed at the same time. There were significant differences for values of ADC_slow_ among hemangiomas, BSTTEHs, and STSs (Table 3). The value of ADC_slow_ decreased from BSTTEHs to STSs with hemangiomas in the middle level. The reason is that potential proliferation of STSs is faster than BSTTs. For STSs, there are large nuclei, less cytoplasm, and a dense array of cells, which lead to the extracellular space reduced, the cell membrane permeability reduced, and the water molecular diffusion limited.^14–17^ So ADC_slow_ decreased significantly. According to ROC curve, the cut-off value of ADC_slow_ without perfusion effects is smaller than that of ADC_standard_ in the bi-exponential of IVIM model. As stated above, ADC_slow_ may be a meaningful parameter for differential diagnosis of hemangiomas, BSTTEHs, and STSs.

Microcirculation perfusion, namely fast moving component in the bi-exponential of IVIM model, is more sensitive to the MR signal attenuation.^18^ According to the bi-exponential theory, ADC_fast_ is closely related to microvessel density of tumor tissues at low b value (<200 s/mm^2^). In this study, ADC_fast_ was significantly greater than related ADC_slow_. It means that ADC_fast_ is sensitive to MR signal attenuation at lower b values. There is a maximum value of ADC_fast_ of STSs as the same results of report^12^ about ADC_fast_ for different groups (cervical tumor, myometrium, and leiomyoma), which confirmed ADC_fast_ associated with the degree of tissue microvessel perfusion.

The fractional volume of capillary blood flowing in each voxel is measured using f.^19^ f value may correlate with the amount of normal angiogenesis with intact vessels in terms of basement membrane thickness and pericyte coverage, and it increases with the augmented tissue perfusion components.^20^ Our results suggest that hemangiomas (0.682 ± 0.060) are rich in capillaries per unit tumor volume, while STSs (0.503 ± 0.068) are relatively poor in capillaries because liposarcomas and extraskeletal myxoid chondrosarcomas are not rich in blood vessels. Therefore, f value may be an indicator of intact vascular permeability.

There are several limitations in this research. First of all, a major limitation of the present study was the small patient cohort of different STTs. Furthermore, IVIM imaging is sensitive to little movement, which may be problematic for thigh IVIM imaging due to pulsation of arteries. Lastly, this study was only to screen out hemangioma from BSTTs, and further research is necessary to differentiate other types of STTs.

## CONCLUSION

To our knowledge, this is the first of its kind report about the application of pixel-based IVIM imaging to evaluate heterogeneous STTs. It may provide useful information to discriminate malignant tumors from benign ones regarding patient stratification and strategy in further treatment.
